# *Oxalis erythrorhiza* Gillies ex Hooker et Arnott (Oxalidaceae): Chemical Analysis, Biological In Vitro and In Vivo Properties and Behavioral Effects

**DOI:** 10.3390/antiox13121494

**Published:** 2024-12-07

**Authors:** Jessica Gómez, Mario J. Simirgiotis, María Sol Kruse, Carlos Gamarra-Luques, Beatriz Lima, José Zaragoza, Mauricio Piñeiro, Alejandro Tapia, Héctor Coirini, Mariana Rey

**Affiliations:** 1 Instituto de Biotecnología-Instituto de Ciencias Básicas, Departamento de Ingeniería Agronómica, Universidad Nacional de San Juan (UNSJ), San Juan J5400ARL, Argentina; jegomez@unsj.edu.ar (J.G.); blima@unsj.edu.ar (B.L.); josedanielzaragoza@gmail.com (J.Z.); mauridpg@gmail.com (M.P.); atapia@unsj.edu.ar (A.T.); 2 Consejo Nacional de Investigaciones Científicas y Técnicas (CONICET), Ciudad Autónoma de Buenos Aires C1425FQB, Argentina; sol.kruse@conicet.gov.ar (M.S.K.); cgamarraluques@gmail.com (C.G.-L.); hcoirini@ibyme.conicet.gov.ar (H.C.); 3 Instituto de Farmacia, Facultad de Ciencias, Campus Isla Teja, Universidad Austral de Chile, Valdivia 5090000, Chile; 4 Center for Interdisciplinary Studies on the Nervous System (CISNe), Universidad Austral de Chile, Valdivia 5090000, Chile; 5 Laboratorio de Neurobiología, Instituto de Biología y Medicina Experimental (IBYME-CONICET), Ciudad Autónoma de Buenos Aires C1428ADN, Argentina; 6 Instituto de Medicina y Biología Experimental de Cuyo Universidad Nacional de Cuyo (IMBECU), CCT CO NICET Mendoza, Mendoza M5500IRA, Argentina

**Keywords:** UHPLC-ESI-QTOF-MS analysis, antioxidant, anti-inflammatory, cytotoxicity, open field, elevated plus maze, novel object location

## Abstract

In this work, a decoction (DOe) and a methanolic global extract (MGEOe), obtained with the aerial parts of *Oxalis erythrorhiza* Gillies ex Hooker et Arnott (Oxalidaceae), were evaluated. The high-resolution liquid chromatography in conjunction with electrospray ionization quadrupole time-of-flight mass spectrometry (UHPLC-ESI-QTOF-MS) analysis showed forty compounds in MGEOe and twenty-nine in DOe, including flavones, C-glycosyl flavones, isoflavones, fatty acids, terpenes, phenolic acids, and sterols. The antioxidant properties were evaluated by DPPH, TEAC, FRAP, and ILP assays. Both DOe and MGEOe showed stronger antioxidant activities. The anti-inflammatory effects were evaluated by COX inhibition method, where DOe demonstrated a significant inhibitory effect. The cytotoxic effects were evaluated in the tumoral HCT-116 and non-tumoral HBL-100 cell lines, revealing a selective action from DOe and MGOe on cancer cells. DOe was evaluated in an animal model of insulin resistance, which is characterized by alterations in glucose and lipid metabolism, as well as cognitive impairments, including anxiety-like behavior and memory deficits. Male SD rats received sucrose (10% *w*/*v*, SUC), a half dilution of DOe (5% *w*/*v*) with sucrose (HDOeS) or DOe with sucrose (DOeS) from PND21 to PND61. Then, anxiety-like behavior and spatial memory were assessed using the open field (OF), elevated plus maze (EPM) and the novel object location (NOL) tests, respectively. Serum parameters basal glycemia, total cholesterol (TC) and tryglicerides were measured using commercial kits. The lipid peroxidation was determined in homogenates of cerebral cortex, hippocampus and hypothalamus by TBAR assay. Only HDOeS exhibited lower anxiety-like behavior in OF and improved performance in NOL compared to SUC. Furthermore, DOeS showed reduced serum parameters, while HDOeS presented lower TC levels than SUC. No differences were observed on TBAR assay. The beneficial properties of these preparations could be attributed to the identified metabolites. These findings highlighted *O. erythrorhiza* as a potential source of compounds to improve human health; however, further research is required to elucidate its mechanisms of action.

## 1. Introduction

Plants not only fulfill essential ecological roles but also serve as an abundant source of inspiration for innovations in green nanotechnology, biomedicine, and architecture. In particular, phytomedicine research holds significant value in the biomedical field, offering the potential to uncover novel therapeutic agents derived from natural sources [1]. For centuries, plants have been central to traditional medicine, providing bioactive compounds with diverse pharmacological properties, including anti-inflammatory, antioxidant, and anticancer effects. Phytomedicine bridges the gap between traditional knowledge and modern biomedical applications, enabling the discovery and development of safer, more cost-effective treatments. Furthermore, studying the mechanisms of action of plant-derived compounds can inspire innovative approaches to addressing complex health challenges, such as chronic diseases and drug resistance. Advancing research in this field is therefore essential for expanding the range of biomedical tools and enhancing global healthcare outcomes.

In the central Andean regions of the province of San Juan of Argentina, there is a significant number of plants used in popular medicine to treat several health disorders. The Andean cushion herb *Oxalis erythrorhiza* Gillies ex Hooker et Arnott (Oxalidaceae), commonly known as “boldo de la cordillera”, is employed in the treatment of hepatic and heart complaints even in the absence of scientific support [2]. This plant is a perennial shrub that forms very dense cushions, often exceeding 30 cm in diameter. Its rhizomes and woody stems are dark at the base, while its petioles, peduncles, and branches are reddish. These branches are covered by the stipules of the leaves, which consist of three narrow, obcordate, and hairy leaflets measuring 1 to 2 mm. The flowers are yellow and solitary, with five petals ranging from 6 to 8 mm, barely protruding above the foliage. The fruit is a small globose capsule, and its seeds are ovoid-asymmetric, measuring 1.2 mm in length, sharp, brown, and marked with interrupted transverse striations. This plant thrives in high mountain habitats, growing at altitudes between 1800 and 4000 m.a.s.l. on the eastern slopes of the Andes [3]. In the literature, there are few reports on the chemistry and biological activity of *O. erythrorhiza*. The hexane and dichloromethane (DCM) extracts of *O. erythrorhiza* showed activity against dermatophytes and bacteria [4]. So far, the literature only reports the isolation of embelin and alkyl phenols from DCM extracts [5]. Other reports state that extracts of Oxalidaceae family members produce antioxidant, cytotoxic and anti-inflammatory effects [6,7,8], improve parameters like basal glycemia (BG), total cholesterol (TC) and triglycerides (TG) [9,10,11] and produce beneficial effects on anxiety behavior and memory [12,13]. However, these latter biological properties of extracts and decoction are poorly explored in *O. erythrorhiza*. On the other hand, although various drugs are used to treat different health impairments, many produce side effects or lack therapeutic action in certain patients, and represent high costs for health systems. That is why the search for improved and low-cost medicinal treatments remains in force. Taking into account this aspect and the fact that the extracts and decoction properties of *O. erythrorhiza* are poorly explored, the aim of this work was to update the chemical profile, the antioxidant, anti-inflammatory and cytotoxic properties in vitro, along with the hypoglycemic, hypolipidemic and anxiolytic properties in vivo. This is the first study to investigate a decoction similar to that traditionally used by the local population, with the aim of supporting the medicinal application of this Andean species while also exploring its potential biological activities.

## 2. Materials and Methods

### 2.1. Chemicals

Ultra-pure water, with total organic carbon (TOC) levels below 5 µg/L, was sourced using an Arium 126 61316-RO purification system coupled with an Arium 611 UV unit (Sartorius, Goettingen, Germany). High-purity methanol (HPLC grade) and mass spectrometry-grade formic acid were supplied by J. T. Baker (Phillipsburg, NJ, USA), while HPLC-grade chloroform was provided by Merck (Santiago, Chile). Various drugs and HPLC standards, including gallic acid, quercetin, catechin, orientin, isoorientin, isovitexin, and rutin, among others, were acquired from Sigma-Aldrich Chem. Co. (St Louis, MO, USA) or Extrasynthèse (Genay, France).

### 2.2. Plant Material, Decoction (DOe) and Methanolic Extract (MGEOe)

The aerial parts of *O. erythrorhiza* were collected from the Bauchaceta Mountains in San Juan Province, Argentina, during the blossoming period (December–February 2022) at an altitude between 4000 and 4500 m.a.s.l. The entire plants were dried at room temperature (25 °C) and stored in conditions devoid of light and heat. A voucher specimen had been previously deposited in the herbarium of the Escuela de Química y Farmacia, Universidad de Chile (SQF), Santiago, Chile, under the reference number SQF 21009.

The DOe was prepared at 10% *w*/*v* with dried and milled leaves of the plant and water purified with PSA equipment. After 30 min of boiling, the decoctions were filtered and then stored at −20 °C until the animal treatments. Representative samples of each decoction (100 mL) were lyophilized with LA-B3 RIFICOR equipment (Buenos Aires, Argentina; yield: 1.13% *w*/*w*). The samples were stored in a freezer at −40 °C until the UHPLC-ESI-QTOF-MS analysis, phenolic and flavonoid quantification, and in vitro and in vivo assays.

The MGEOe was obtained by reflux extraction from the air-dried, ground aerial parts of the plant (1 kg) using methanol (3000 mL) in three separate 30 min intervals at 60 °C. The resulting mixtures were concentrated under reduced pressure using a Yamato rotary evaporator (RE-300-AW2, Santa Clara, CA, USA). The final MGEOe product (200 g, yielding 20% *w*/*w* relative to the initial dry material) was stored at −20 °C until it was analyzed via UHPLC–PDA–QTOF-MS, as well as for the quantification of phenols and flavonoids, and in vitro assays.

### 2.3. Ultra-High-Resolution Liquid Chromatography Analysis (UHPLC-ESI-QTOF-MS)

The separation and identification of secondary metabolites in DOe and MGEOe were performed following previous studies [14,15], utilizing a UHPLC-ESI-QTOF-MS system. This system included a UHPLC Ultimate 3000 RS with Chromeleon 6.8 software (Dionex GmbH, Idstein, Germany) and a Bruker maXis ESI-QTOF-MS. Mass spectrometry data were processed using Bruker Data Analysis 4.0 software (Bruker Daltonik GmbH, Bremen, Germany) and ACD Lab Spectrum Processor (New York, NY, USA), with sodium formate as the internal standard.

### 2.4. The Total Phenol and Flavonoid Content

The total phenol content and the flavonoid content were expressed as milligrams of gallic acid equivalents (GAE) or milligrams of quercetin equivalents (QE) per gram of DOe and MGEOe (mg GAE/g DOe or MEGOe), respectively [16]. The values were obtained in quadruplicate using a Multiskan FC Microplate Photometer (Thermo Scientific, Waltham, MA, USA). The results are expressed as mean ± SD.

### 2.5. In Vitro Studies

#### 2.5.1. Antioxidant Activity

##### Radical Scavenging Capacity Assay of 2,2-Diphenyl-1-picrylhydrazyl (DPPH)

The free radical scavenging activity of DOe and MGEOe was assessed using the DPPH assay. The concentration required to achieve 50% radical scavenging (EC50) was determined from a graph plotting inhibition percentage at 517 nm against concentrations of DOe and MGEOe. Catechin (Sigma-Aldrich, ≥98%, St. Louis, MO, USA) served as the reference compound (EC50 4.1 μg/mL). Each test was conducted in quadruplicate [16].

##### Ferric-Reducing Antioxidant Power Assay (FRAP)

Results from the FRAP assay were calculated using linear regression based on the FRAP–Trolox calibration curve and are expressed as milligrams of Trolox equivalents per gram of DOe or MGEOe (mg Trolox/g DOe or MGEOe). All tests were conducted in quadruplicate [16].

##### Trolox Equivalent Antioxidant Activity Assay (TEAC)

The TEAC assay results were obtained through linear regression from a calibration curve created using Trolox concentrations ranging from 0 to 1 mM and are reported as milligrams of Trolox equivalents per gram of DOe and MGEOe (mg Trolox/g DOe or MGEOe). Each test was performed in quadruplicate [16].

##### Inhibition of Lipid Peroxidation (ILP) in Erythrocytes

The ILP assay in erythrocytes was performed following the previously described methodology [16], employing two concentrations of DOe and MGEOe (100 and 250 µg/mL). Catechin was employed as positive control (70% of ILP at100 µg/mL). The obtained results in quadruplicate were expressed as percentages of ILP.

#### 2.5.2. Anti-Inflammatory Effects: Cyclooxygenase (COX) Inhibition Method

The inhibitory activity of DOe (18; 36 and 72 µg/mL) on COX-1 (ovine) and COX-2 (human) was determined using a commercial colorimetric kit 701050 (COX Colorimetric Inhibitor Screening Assay Kit, Cayman Chemical Co., Ann Arbor, MI, USA) following the manufacturer’s instructions, which involved measuring prostaglandin (PG) levels by ELISA. Diclofenac sodium (1–2 µg/mL) and etoricoxib COX-2 specific inhibitor (2.5–5.0 µg/mL) were used as a positive control. Once the buffer, hemin and enzymes were added to the background wells, 100% initial activity wells (Abs COX) and inhibitor wells, they were incubated for 5 min at 25 °C. Then, the colorimetric substrate was added to all the wells and arachidonic acid was quickly added. Finally, the solution was incubated for 2 min at 25 °C and measured for absorbance at 590 nm using a Multiskan FC Microplate Photometer (Thermo Scientific, Waltham, MA, USA). The results are expressed as mean ± SD. The obtained results were expressed as percentage of inhibition [17,18] of COX-1 and COX-2 enzymes, defined as:

% of Inhibition = ((A − B)/A) × 100(1)

A = (Abs100% initial Act − Abs background); B = (Abs inhibitor − Abs background)

#### 2.5.3. Antitumoral and Cytotoxic Assays

##### Cell Culture Procedure

Two different human cell lines were used: HCT-116, representative of colorectal cancer, and HBL-100, considered as non-tumoral epithelial cells. Both cell lines were obtained from the American Type Culture collection (ATCC, Manassas, VA, USA). The cells were cultured as a monolayer in a 37 °C humidified atmosphere enriched with 5% of CO_2_ in Dulbecco’s Modified Eagle Medium (DMEM, Gibco, Sigma-Aldrich Chem. Co. St Louis, MO, USA; pH 7.6), supplemented with 10% (*v*/*v*) fetal bovine serum (FBS, Internegocios, Mercedes, Buenos Aires Province, Argentina), 3.7 g/L NaHCO_3_ (Sigma-Aldrich, USA) and antibiotics (100 IU/mL penicillin and 100 µg/mL streptomycin, Gibco, Waltham, MA, USA).

##### Methyl Thiazolyl Tetrazolium (MTT) Assay

Cell proliferation was assessed by the MTT colorimetric assay as described previously [16]. Briefly, 3.5 × 10^4^ cells/100 µL were seeded in a 96-well microplate and let to attach overnight. Cells were treated with Doe (125, 250, 500, 1000 and 2000 µg/mL) and/or MGEOe (62.5, 125, 250, 500 and 1000 µg/mL) for 48 h. The stock solutions (100 mg/mL) of Doe and MGEOe were prepared in water or dimethyl sulfoxide (DMSO), respectively. The highest concentration of MGEOe was determined by DMSO toxicity. Solvent presence in concentrations higher than 1000 µg/mL resulted in intrinsic cytotoxicity. Thereafter, the medium was replaced with MTT solution (0.5 mg/mL) in DMEM lacking phenol red, FBS, and antibiotics. After 4 h incubation in the dark, the solution was removed, and 100 µL of DMSO per well was added to dissolve the formazan crystals. The optical density was measured at 570 nm with a Thermo Scientific Multiscan Elisa reader. The median effect dose (Dm), obtained with CompuSyn 1.0 software [19], was used as indicative of extract potency. All assays were performed three times in triplicate.

### 2.6. Experimental Procedure In Vivo

Adult male Sprague-Dawley rats (PND 21; *n* = 60) were maintained under standard laboratory conditions within a temperature- and humidity-controlled vivarium, adhering to a 12 h light/dark cycle with ad libitum access to food and water. All procedures related to animal care and use were conducted in accordance with the European Community Council Directive (86/609/EEC) and the National Institutes of Health Guide for the Care and Use of Laboratory Animals, and were approved by the ethical committee of IBYME (resolution number 021/2021) in CABA, Argentina. The animals were fed with a normal diet consisting of 3.3 kcal/g, with the following composition: protein (18.2%), carbohydrates (56.9%), lipids (3.9%), vitamins and minerals (3%), fiber (5%), and moisture (13%) (sourced from Gepsa feeds, Grupo Pilar SA, Buenos Aires Province, Argentina). The animal model was developed as described previously [20] with modifications. Briefly, from PND 21, the animals received water (CON, *n* = 10), sucrose 10% *w*/*v* (S, *n* = 10, commercial sugar, Ledesma, Ingenio Ledesma S.A.A.I, Buenos Aires, Argentina), a half dilution of Doe (5% *w*/*v*) alone (HDOe, *n* = 10) or with sucrose 10% *w*/*v* (HDOeS, *n* = 10), or Doe alone (*n* = 10) or with sucrose 10% *w*/*v* (DoeS, *n* = 10) for 40 days. The groups CON, HDOe and Doe were developed to evaluate the treatment effects in the normal condition. Their comparisons and comparisons between SUC and CON are in the Supplementary Materials. The body weight (BW) of the animals was recorded at the beginning (PND21) and at the end (PND61) of the treatments. The BW gain was calculated by subtracting the BW at PND21 from the BW at PND61. Beverage consumption was recorded every other day between 09:00 and 10:00 a.m. for 40 days of treatment, inside each group. At PND 61, all beverages were replaced by water. After one day, the animals were evaluated with a glucose tolerance test (GTT). The behavioral tests were performed between PND 63 and 69. After this period, the animals, fasted for 6 h, were rendered unconscious using CO_2_ and then decapitated. Blood was collected from the trunk to assess lipid parameters. The hypothalamus, hippocampus and cerebral cortex were dissected, frozen and stored at −80 °C [21,22] to determine the degree of lipid peroxidation by thiobarbituric acid reactive substances (TBAR) assay. The determination of BW gain, the beverage volume, the GTT, the serum parameters and TBAR were conducted on 7 animals from each group from two independent assays (*n* = 3 or 4/assay).

#### 2.6.1. Determination of GTT and Serum Parameters

The BG was determined in a drop of blood, obtained from the tail vein of the 6 h fasted animal, using a test strip and the “One Touch Ultra” glucometer (Johnson & Johnson^®^ Plaza, New Brunswick, NJ, USA). The GTT and the area under curve (AUC) were obtained as described previously [20]. The TC and TG levels were determined in the serum obtained from the trunk blood, as described previously [22,23].

#### 2.6.2. TBAR Assay Method

The homogenates of hypothalamus, hippocampus and cerebral cortex were obtained following the protocol described previously [21,22]. The protein concentration was determined with the spectrophotometric method described by [24]. The extent of lipid peroxidation in these homogenates was evaluated by measuring the production of TBAR, which indicates the presence of malondialdehyde (MDA) in the tissue, according to the previously described protocol [20].

#### 2.6.3. Behavioral Tests Procedure

All the tests were performed as described previously [20]. All devices were illuminated by a 42 W light positioned 100 cm above, directed towards the ceiling, ensuring uniform light intensity across all areas. The animals were placed in the testing room and allowed to acclimate for at least 15 min before testing. To eliminate olfactory cues, the devices were thoroughly cleaned with a 70% ethanol solution after each trial. All tests were recorded on video and analyzed using ANY-maze Video Tracking System version 7.44 (Stoelting Co., Wood Dale, IL, USA) by two observers who were blind to the treatments administered to the animals.

##### Open Field and Elevated Plus Maze Tests

The open field (OF) was digitally partitioned into zones: central, middle, and peripheral, while the elevated plus maze (EPM) was divided into central, open, and closed arms. The number of entries, distance traveled, and time spent in each zone were analyzed. The events of rearing [25] and grooming [26] were also analyzed. Additionally, the head dip events (when the animals protrude the head over the edge of an open arm) were determined in the elevated plus maze as a measure of risk assessment [27,28,29,30].

##### Novel Object Location Test

The novel object location test (NOL) consists of two trials. In the first trial (T1), the animal is placed in a device (a wooden box with an open top and dark interior) with two identical objects positioned equidistantly for 5 min. After a 2 h intertrial interval, the second trial (T2) is conducted, where the animal is placed back in the device with one object relocated to a new position (N), while the other remains in its original, familiar location (F). The animal explores the objects for 5 min. The time spent exploring each object is recorded and it is important to note that the animal should explore each object for 20 s to be considered for the analysis. The results are presented as the exploration ratio (ER), calculated as the time spent exploring object N divided by the total time spent exploring both objects [tN/(tN + tF)]. An ER of 0.50 or lower indicates either an equal preference for objects N and F or a preference for F, suggesting potential memory impairment. To ensure that the animals traveled the same distance within each trial, the number of virtual lines crossed was also analyzed.

#### 2.6.4. Statistical Analysis

The results were analyzed with GraphPad Prism (GraphPad Software Inc., v.9, San Diego, CA, USA). The comparisons between two groups were conducted using Student t test, whereas the comparisons between three groups were assessed using one-way ANOVA followed by Duncan or Newman–Keuls post hoc tests. All results are expressed as mean ± standard deviation (SD). All differences were considered significant when *p* < 0.05.

## 3. Results

### 3.1. Metabolite Profiling: UHPLC-ESI-QTOF-MS Analysis of Doe and MGEOe

**Figure 1:**
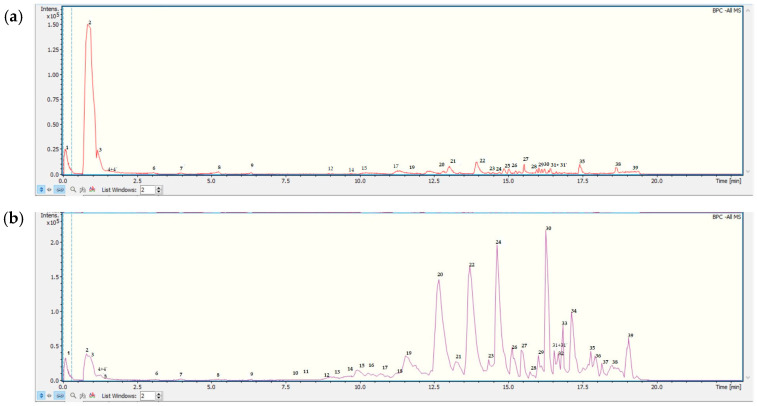
UHPLC Q-TOF-TIC chromatogram of DOe (**a**) and MGEOe (**b**). The peak numbers correspond to internal standard Na formiate (**1**); monohexosyldiglyceride (**2**); quinic acid (**3**); vicenin II (apigenin 6,8-di C-B-D-glucopiranoside) (**4**); chlorogenic acid (**4**′); luteolin-6-C-glucoside (isoorientin; (**5**); bis-3-(4′,5,7-trimethoxy-flavanone)-5′-(2′,4,4′-trimethoxy-chalcone) (**6**); rutin (**7**); isovitexin (**8**); meliartenin (**9**); 4′,5,7-trihydroxy-3,6-dimethoxyflavone (**10**); 8-hydroxy-5-methoxyflavanone (**11**); 17-hydroxylinolenic acid (**12**); diffractaic acid (**13**); 9-HPODE octadecenedioic acid (**14**); 9-hydroxy-10,12-octadecadienoic acid (**15**); 13-HPODE octadecenedioic acid (**16**); (9Z,12R)-12-hydroxyoctadec-9-enoic acid (**17**); ricinoleic acid (**18**); 5-(octadecyloxy) isophthalic acid (**19**); ricinoleic acid isomer (**20**); lesquerolic acid (**21**); pinolenic acid (**22**); 9-octadecenoic acid (Z)-,2-hydroxyethyl ester (**23**); roccellaric acid (**24**); alpha-boswellic acid (**25**); eicosapentaenoic acid (**26**); pristimerin (**27**); dodecanoic acid,2-ethylhexanoic acid, propane-1,2,3-triol (**28**); palmitic acid (**29**); FAHFA 18:1/2:0 hydroxy fatty acid ester: icos-10-enedioic acid (**30**); 22-oxodocosanoic acid (**31**); PI 34:21-phosphatidyl-1D-myo-inositol (**31**′); isopropyl linoleate (**32**); PI(16:0/13-HODE) hydroxyoctadecadienoic acid (**33**); muricatenol (**34**); 28-O-acetylbetulin-3-yl-β-D-glucopyranoside (**35**); 3β-O-acetyl-28-O-lup-20(29)-ene (**36**); 1-stearyl-2-cholesterylcarbonoyl-3-trityl glycerol (**37**); aipolic acid(**38**); daucosterol (**39**).

**Figure 2:**
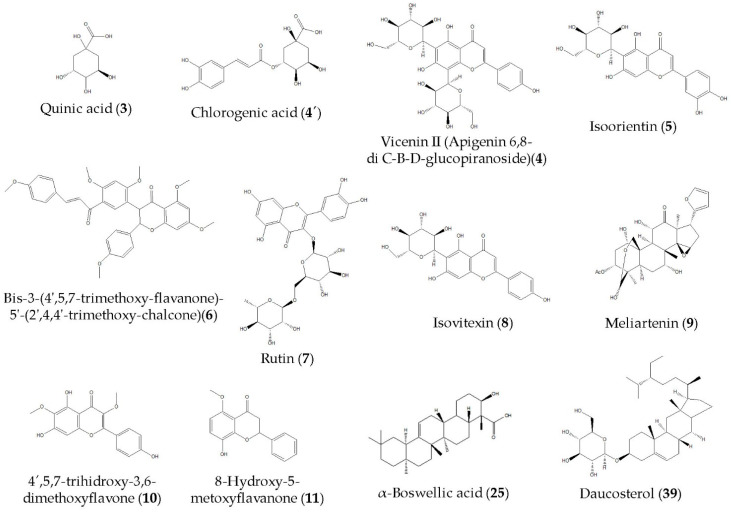
Molecular structures of main compounds identified in DOe and MGEOe.

The exhaustive UHPLC-ESI-QTOF-MS analysis in negative mode revealed the presence of thirty-eight peaks in MGEOe, corresponding to forty compounds, and twenty-seven peaks corresponding to twenty-nine compounds in Doe. These included C-glycosyl flavones, flavones, aromatics, terpenes, sterols, furans, and several fatty acids; all of them are tentatively identified for the first time in *O. erythrorhiza*. The identification strategy implemented involved spiking experiments with available standards. Additionally, a comprehensive access strategy to several databases, including MONA mass spectrometry and Metaboscape, was employed, along with the use of specific software such as Data Analysis 4.0 (Bruker Daltonik GmbH, Bremen, Germany) and ACD Lab Spectrum Processor (Toronto, Canada). Figure 1 and Table 1 display the high-resolution UHPLC–PDA–QTOF analysis results for metabolite identification in Doe and MGEOe. Additionally, Figure 2 shows the chemical structures of some of the main compounds identified in Doe and MGEOe.

### 3.2. In Vitro Studies

#### 3.2.1. Total Phenolic and Flavonoid Contents, Antioxidant Activity and Inhibition Enzyme COX of DOe and MGEOe

Both extracts exhibited high levels of total phenolics, measuring 69 mg GAE/g for DOe and 65 mg GAE/g for MGEOe. Also, the flavonoid contents were 43 mg QE/g DOe and 31 mg QE/g MGEOe (Table 2). Compared to catechin, quercetin and gallic acid (value range EC_50_: 1–10 µg/mL), the antioxidant activity in the DPPH assay was moderate for both extracts (DOe EC_50_: 70 µg/mL and MGEOe EC_50_: 62 µg/mL). DOe and MGEOe exhibited a significant ILP in erythrocytes, reaching 81–83%, respectively, at 250 μg DOe or MGEOe/mL (Table 2), compared to positive control catechin, which showed 70% of ILP at 100 µg/mL. Regarding enzyme inhibition of COX-1 and 2, DOe showed a good percentage of inhibition of COX-2 (86.97% at 72 µg/mL).

#### 3.2.2. Cytotoxicity Study of DOe and MGEOe

**Figure 3:**
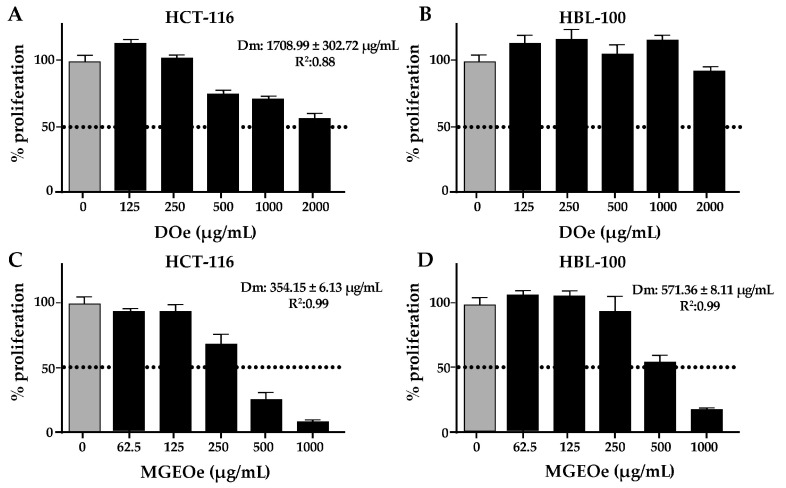
HCT-116 and HBL-100 cell proliferation after 48 h treatment with DOe (**A**,**B**) or MGEOe (**C**,**D**) and their respective Dm values.

DOe produced a slight cytotoxic effect on HCT-116, but not on HBL-100 (Figure 3A,B). The dose–response effect against proliferation was present (R^2^ = 0.88), but the maximum dose was not enough to reach the Dm. The estimated Dm value was 1708.99 ± 302.72 µg/mL. In contrast, MGEOe induces marked cytotoxic effects in both cell lines. The dose–response effects against proliferation were present in both cell lines (R^2^ = 0.99). The Dm estimated was 354.15 ± 6.13 µg/mL for HCT-116 (Figure 3C) and 571.36 ± 8.11 µg/mL for HBL-100 (Figure 3D). The comparison between both Dm values revealed significant differences, revealing specific cytotoxic effects on HCT-116 (*p* = 0.002). The comparison between the extracts revealed that MGEOe showed higher potency than DOe on HCT-116 (*p* = 0.0015).

### 3.3. In Vivo Study

#### 3.3.1. BW Gain and Average Beverage Volume

**Figure 4:**
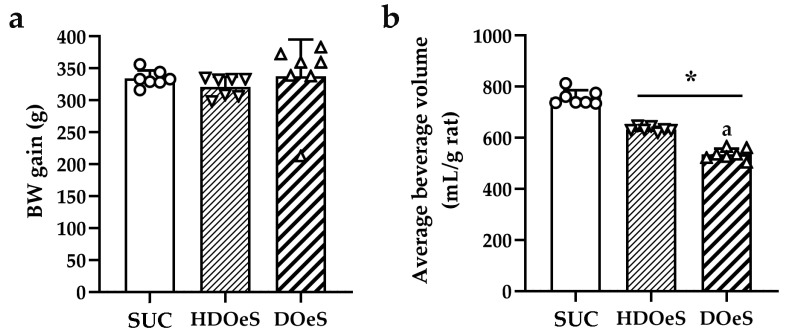
BW gain (**a**) and average beverage volume (**b**) of SUC (empty circles), HDOeS (empty inverted triangles) and DOeS (empty triangles). Results are reported as mean ± SD from two independent assays (*n* = 7 animals per group). Significant differences were assessed using one-way ANOVA (F_BWgain_(2,18) = 0.464; *p* = 0.6359 and F_Beverage_(2,18) = 181.303; *p* < 0.0001) followed by Newman–Keuls post hoc test. * and ^a^ *p* < 0.05; * refers to SUC and ^a^ refers to HDOeS.

The analysis of BW gain did not show any significant differences between HDOeS, DOeS and SUC (Figure 4a). Regarding to the average beverage volume, HDOeS and DOeS drank less beverage than SUC (16.35% and 29.21%, respectively; *p* < 0.0001). Also, DOeS drank less beverage than HDOeS (15.39%; *p* < 0.0001 Figure 4b).

#### 3.3.2. GTT and Serum Parameters

**Figure 5:**
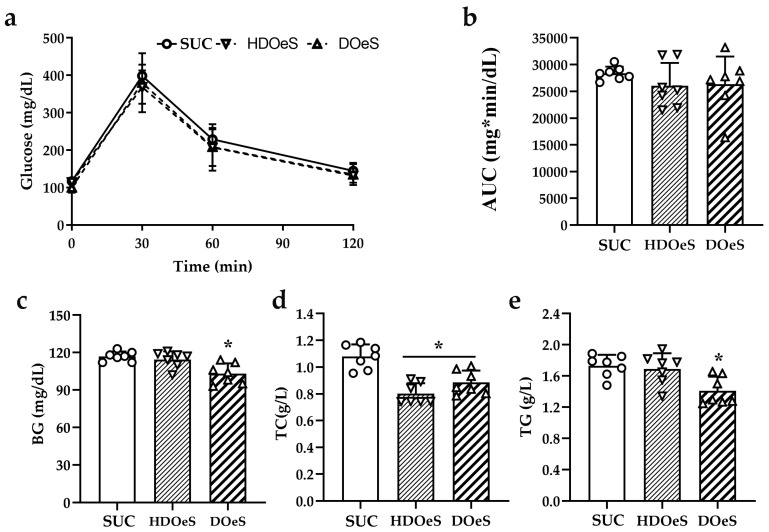
Levels of glucose during the GTT (**a**) and its AUC values (**b**), levels of BG (**c**), TC (**d**) and TG (**e**) after 40 days of administration of SUC, HDOeS and DOeS. Results are expressed as mean ± SD from 2 independent assays (*n* = 7 animals/group). Significant differences were determined by one-way ANOVA (F_AUC_(2,18) = 1.348; *p* = 0.2847; F_BG_(2,18) = 7.603; *p* = 0.0040; F_TC_(2,18) = 5.864; *p* = 0.0109 and F_TG_(2,18) = 6.868; *p* = 0.0061) followed by Newman–Keuls post hoc test. *p* < 0.05, * refers to SUC.

The analysis of AUC did not show significant differences between the groups (Figure 5a,b). No significant differences were observed in the levels of BG of HDOeS and SUC. In contrast, DOeS presented a decrease in BG levels compared to SUC (14.67%; *p* < 0.01) and HDOeS (12.86%; *p* < 0.001; Figure 5c). The levels of TC in HDOeS and DOeS were lower than those of SUC (17.44% and 11.69%, respectively; *p* < 0.05). Also, no significant differences were observed between HDOeS and DOeS (Figure 5d). The analysis of TG levels did not show significant differences between the HDOeS and SUC groups. However, DOeS presented lower levels than SUC and HDOeS (18.44% and 16.61%, respectively; *p* < 0.01; Figure 5e).

#### 3.3.3. TBAR Assay

The analysis did not reveal significant differences between HDOeS and DOeS and SUC in any of the tissues evaluated (Table 3).

#### 3.3.4. Behavioral Tests

##### OFT and EPM

**Figure 6:**
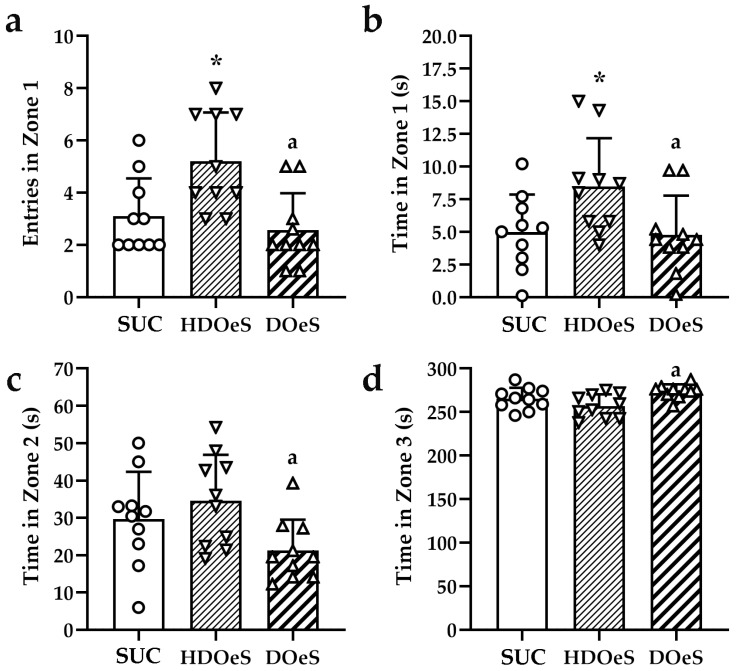
Entries in zone 1 (**a**), time spent in zones 1 (**b**), 2 (**c**) and 3 (**d**) in the OFT after 40 days of administration of SUC (empty circles), HDOeS (empty inverted triangles) and DOeS (empty triangles). Results are presented as mean ± SD from two independent assays (*n* = 10 animals per group). Significant differences were assessed using one-way ANOVA (F_Entrieszone1_(2,27) = 7.642; *p* = 0.0023; F_Timezone1_(2,27) = 4.165; *p* = 0.0265; F_Timezone2_(2,27) = 3.576; *p* = 0.0419 and F_Timezone3_(2,27) = 5.349; *p* = 0.0110) followed by Newman–Keuls post hoc test. *, ^a^ *p* < 0.05, * refers to SUC and ^a^ refers to HDOeS.

In the OFT, the analysis of total distance and lines crossing did not show significant differences between the groups (F_Totaldistance_(2,27) = 0.646; *p* = 0.5322; F_Lines_(2,27) = 0.822; *p* = 0.4504). In the zone 1, HDOeS entered more and spent more time than SUC (67.74%; *p* < 0.001 and 70.42%; *p* < 0.05, respectively, Figure 6a,b). However, the performance of DOeS was similar to SUC. Also, DOeS entered less and spent less time in the zone than HDOeS (50.77%; *p* < 0.001 and 43.57%; *p* < 0.05, respectively, Figure 6a,b). In zones 2 and 3, no significant differences were observed between the groups (Figure 6c,d). However, HDOeS spent less time in zone 2 (38.55%; *p* < 0.05) and more time in zone 3 (6.63%; *p* = 0.05) than HDOeS (Figure 6c,d). The analysis of the remaining parameters within each zone did not reveal significant differences between the groups (F_Distancezone1_(2,27) = 2.277; *p* = 0.1219; F_Entrieszone2_(2,27) = 2.388; *p* = 0.1110; F_Distancezone2_(2,27) = 2.185; *p* = 0.1319; F_Entrieszone3_(2,27) = 0.944; *p* = 0.4016 and F_Distancezone3_(2,27) = 2.193; *p* = 0.1311).

The analysis of grooming behavior did not indicate significant differences in either the number of events or the duration of the activity (F_Events_(2,22) = 2.201; *p* = 0.1344 and F_Time_(2,22) = 0.081; *p* = 0.9222). It is important to note that only half of HDOeS performed the activity. Similarly, the analysis of rearing did not reveal significant differences in the number of events and/or time spent on the activity (F_Events_(2,27) = 0.119; *p* = 0.8886 and F_Time_(2,27) = 2.154; *p* = 0.1356).

In the EPM, the analysis did not reveal significant differences between the groups in any of the parameters evaluated in closed (F_Entries_(2,27) = 2.536; *p* = 0.0979; F_Time_(2,27) = 0.712; *p* = 0.4996 and F_Distance_(2,27) = 0.588; *p* = 0.5625) and open arms (F_Entries_(2,27) = 3.218; *p* = 0.0558; F_Time_(2,27) = 2.312; *p* = 0.1183 and F_Distance_(2,27) = 2.312; *p* = 0.1183), and center (F_Entries_(2,27) = 2.815; *p* = 0.776; F_Time_(2,27) = 0.847; *p* = 0.4398 and F_Distance_(2,27) = 1.059; *p* = 0.3608). The activities of grooming and rearing were only observed in the closed arms. The analysis did not reveal significant differences in grooming (F_Events_(2,22) = 0.691; *p* = 0.5114 and F_Time_(2,22) = 3.118; *p* = 0.0642) and rearing (F_Events_(2,27) = 0.079; *p* = 0.9246 and F_Time_(2,27) = 1.755; *p* = 0.1921). It is important to note that not all the animals performed grooming (SUC *n* = 7; HDOeS *n* = 9 and DOeS *n* = 9). The analysis of head dip events did not show significant differences among the groups (F_Events_(2,27) = 2.241; *p* = 0.1258).

##### NOL

**Figure 7:**
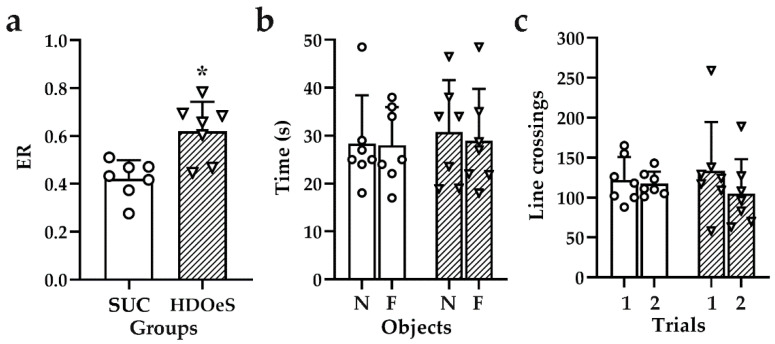
ER (**a**), time dedicated to exploring objects N and F in T1 (**b**) and lines crossing in T1 and T2 (**c**) in NOL test after 40 days of administration of SUC (empty circles) and HDOeS (empty inverted triangles). Results are presented as mean ± SD from two independent assays (*n* = 7 animals per group). Significant differences were assessed using Student’s *t*-test (ER: *p* = 0.0037; time for object N: *p* = 0.6661 and for object F: *p* = 0.8597; and line crossing in T1: *p*= 0.6629 and in T2: *p* = 0.4890). *p* < 0.05, * refers to SUC.

The HDOeS group presented an increase in ER compared to SUC (47.03%; *p* = 0.0037). Also, no differences were observed in the amount of crossed lines in T1 and T2 and the time dedicated to exploring each object in T1 (Figure 7). It is important to note that the DOeS group was excluded from the analysis because only two animals explored both objects for the required duration during the trials.

## 4. Discussion

In this work, forty compounds and twenty-nine compounds were tentatively identified in MGEOe and DOe, respectively, including flavones (**6**, **7**, **10** and **11**), C-glycosyl flavones (**4** and **5**), isoflavone (**8**), fatty acids (**2**, **14**–**18**, **20**–**26**, **28**–**35** and **37**), terpenes (**27**, **38**), phenolic acids (**4′** and **19**), aromatics (**12** and **13**), furane (**9**) and sterols (**36** and **39**), using UHPLC-ESI-QTOF-MS analysis in negative mode. These identifications represent the first report for *O. erythrorhiza*, providing an updated account of the chemical composition of this Andean species. Recent studies have reported the metabolite profiling of various medicinal plants and fruits collected in Argentina and Chile, utilizing two powerful techniques: UHPLC-ESI-OT-MS and UHPLC-ESI-QTOF-MS. These exhaustive analyses have significantly contributed to updating the chemical composition of all the Andean medicinal plants studied, providing a more comprehensive understanding of their bioactive compounds. On the other hand, it has allowed us to provide solid support for some of the evaluated activities by identifying the potential compounds responsible for the biological effects, including the strong antioxidant properties of the decoctions and polar extracts from most of the analyzed species [14,15,16,22,23,31,32,33,34,35].

In the last decade, there has been an increase in research focused on identifying new sources of antioxidant compounds derived from natural products, such as fruits and medicinal plants, in their various forms of consumption. These compounds have the potential to serve as therapeutic options for treating pathological conditions where free radicals play a recognized role [36]. In this study, antioxidant activity was evaluated using assays based on electron transfer mechanisms, including FCR, TEAC, FRAP, and DPPH assays, which can also be neutralized by hydrogen atom donation [36,37]. Among these methods, the DPPH assay is particularly notable for its strengths; it is simple, cost-effective, and rapid, with reproducible results that are comparable to other radical scavenging methods. It is highly sensitive and often shows good correlation with bioactive compounds such as phenols and flavonoids, typically yielding a regression factor R > 0.8. However, these strengths are counterbalanced by certain limitations, including its sensitivity to the presence of Lewis bases, which can affect DPPH absorbance. Additionally, the analysis must be conducted in the dark or protected from light to prevent absorbance decay. Moreover, the absence of DPPH free radicals in the human body limits its physiological relevance [36].

The in vitro studies of MGEOe and DOe revealed significant antioxidant activity, highlighting the effect on ILP process. This could be explained at least in part by the presence of chlorogenic acid (**4**′), isoorientin (**5**), rutin (**7**), isovitexin (**8**), 4′,5,7-trihydroxy-3,6-dimethoxyflavone (**10**), pinoleic acid (**22**), alpha-boswellic acid (**25**), and daucosterol (**39**) among others, which have been reported to have antioxidant activity [38,39,40,41,42,43,44,45,46,47,48,49]. However, the in vivo studies of HDOeS and DOeS did not reveal any significant effects on MDA levels in the tissues evaluated.

The Andean flora has been scarcely investigated for its cytotoxic properties. Recently, extracts and decoctions of two species growing in Argentina have demonstrated cytotoxic potential, supported in part by the comprehensive chemical characterization of these extracts and decoctions [16,35]. The cytotoxicity assays revealed that DOe produced a selective cytotoxic effect against a tumoral cell line, with no effects in non-tumoral cells, whereas MGEOe produced cytotoxic effects in both cell lines. Also, the effect of MGEOe was higher than that of DOe. These cytotoxic effects may be linked to the presence of vicentin II (4), chlorogenic acid (**4**′), isoorientin (**5**), rutin (**7**), meliartenin (**9**), alpha-boswellic acid (**25**), and daucosterol (**39**), which have been reported to have antitumoral properties [38,39,42,49,50,51,52,53,54,55,56].

The analysis of COX inhibition revealed that DOe produced a significant anti-inflammatory effect. This may be related to the presence of certain compounds tentatively identified in the UHPLC-MS analysis, including vicentin II (**4**), chlorogenic acid (**4′**), isoorientin (**5**), isovitexin(**8**), ricinoleic acid (**18**), pinoleic acid (**22**), alpha-boswellic acid (**25**), and daucosterol (**39**), which has reported anti-inflammatory properties [38,40,43,44,45,47,48,49,57,58,59,60,61,62].

On the other hand, the selected in vivo model of insulin resistance is characterized by alterations in glucose and lipid metabolism, oxidative state, as well as cognitive impairments, including anxiety-like behavior and memory deficits [20]. In this work, the administration of HDOeS and DOeS did not modify the BW gain. In contrast, several reports describe that compounds like vicentin II (**4**), chlorogenic acid (**4′**) and rutin (**7**), among others, could modify the BW gain [63,64,65,66,67]. Although these treatments did not modify the response to glucose overload, the animals presented a decrease in BG. This effect may be linked to the presence of compounds such as quinic acid (**3**), chlorogenic acid (**4′**) and bis-3-(4′,5,7-trimethoxy-flavanone)-5′-(2′,4,4′-trimethoxy-chalcone, (**6**), which can play vital functions in the process of regulating glucose metabolism [38,68,69]. Additionally, daucosterol (**39**) may inhibit key glucose metabolism enzymes, helping to maintain blood sugar levels within normal ranges [45]. Regarding the lipid parameters, the administration of the decoction reduces the levels of TC and TG. The hypocholesterolemic properties can be attributed to the combined action of flavonoids, fatty acids, phenolic compounds, and sesquiterpenes, all of which have hypolipidemic effects through several mechanisms [22,23,38,43,48,49,61,70,71,72].

In the evaluation of anxiety levels, only the administration of HDOeS produced an anxiolytic effect, which could be attributed to the action of compounds like chlorogenic acid (**4′**) [73], rutin (**7**) [74], isovitexin (**8**) [75], vicenin II (**4**) [76], ricinoleic acid isomer (**20**) [77], over different pathways. In addition, only the administration of HDOeS improved the spatial memory performance of the animals. This could be attributed to the content of chlorogenic acid (**4′**) [78], rutin (**7**) [79], vicentin II (**4**) [80], daucosterol (**39**) [81], among others, which are capable of improving the cognitive state through different mechanisms. In the animal model reported in this work, the variation in results of the doses employed could be attributed to the difference in the beverage intake. Furthermore, it is important to highlight that administering both doses of decoction to the control animals did not affect their well-being or the evaluated parameters (see Supplementary Materials).

Although the results are promising, additional research is needed to establish whether *O. erythrorhiza* can be regarded as a new source of phytocompounds. The demonstrated antioxidant, anti-tumoral, anti-inflammatory, hypoglycemic, hypocholesterolemic, anxiolytic, and memory-enhancing activities of *O. erythrorhiza* open the way for future studies, including the incorporation of its extracts into polymeric systems, which can preserve the integrity of drugs and biomacromolecules, such as nanoparticles [82,83].

## 5. Conclusions

In summary, the findings presented in this study provide an updated chemical profile of the Andean medicinal species *O. erythrorhiza*. Several of the reported compounds could explain, at least partially, the demonstrated antioxidant, anti-tumoral, anti-inflammatory, hypoglycemic, hypocholesterolemic, anxiolytic and memory improvement activities of *O. erythrorhiza*. While mechanistic and molecular assays are necessary to clarify the action pathways, the current study provides valuable information about this species that may be beneficial in addressing various health conditions. Additionally, to our knowledge, this is the first work that involves the study of *O. erythrorhiza* on in vitro and in vivo assays. Although the results are promising, additional research is needed to determine whether *O. erythrorhiza* can be regarded as a new source of beneficial phytocompounds relevant to human health.

## Figures and Tables

**Table 1:** UHPLC-PDA-Q-TOF identification of metabolites from DOe and MGEOe.

Peak	Tentative Identification	[M-H]-	Retention Time (min)	Measured Mass (*m*/*z*)	Molecular Formula	Accuracy (ppm)	Metabolite Type	MS Ions (*m*/*z*)
**1**	Naformiate (internal standard)	NaC_2_H_2_O_4_	0.12	112.9829	112.9856	3.10	A	
**2**	Monohexosyldiglyceride	C_12_H_18_O_12_	0.78	353.0786	354.0859	17.08	Fatty acid	
**3**	Quinic acid	C_7_H_12_O_6_	0.84	191.0563	192.0636	2.26	Acid	93.0345
**4**	Vicenin II (apigenin 6,8-di C-B-D-glucopiranoside)	C_27_H_30_O_15_	1.20	593.2185	594.2199	2.45	C-glycosil flavone	353.1079
**4′**	Chlorogenic acid	C_16_H_18_O_9_	1.20	353.0886	354.0959	2.45	Phenolic acid	191.0513
**5**	Luteolin-6-C-glucoside (Isoorientin)	C_21_H_20_O_11_	1.50	447.0953	448.1025	3.93	C-glycosil flavone	429.1389 357.1038 327.0886 298.0861
**6**	Bis-3-(4′,5,7-trimethoxy-flavanone)-5′-(2′,4,4′-trimethoxy-chalcone)	C_36_H_34_O_9_	3.13	609.2154	610.2130	3.89	Flavone	447.1456 341.1492 112.9882
**7**	Rutin	C_27_H_30_O_16_	4.42	609.1477	610.1550	4.67	Flavone	301.0333 300.0258
**8**	Isovitexin	C_21_H_20_O_10_	4.48	431.1006	432.1078	5.07	Isoflavone	311.0563 283.0608
**9**	Meliartenin	C_28_H_36_O_10_	6.25	531.2313	532.2236	3.05	Furane	486.2168 385.3007
**10**	4′,5,7-trihydroxy-3,6-dimethoxyflavone	C_17_H_14_O_7_	7.54	329.0677	330.0750	3.31	Flavone	311.1599 255.0120
**11**	8-hydroxy-5-methoxyflavanone	C_16_H_14_O_4_	7.93	269.0817	270.0894	4.06	Flavone	254.0580
**12**	17-hydroxylinolenic acid	C_18_H_30_O_3_	8.45	293.2134	294.2207	4.00	Aromatic	439.0996 311.1636
**13**	Diffractaic acid	C_20_H_22_O_7_	9.62	373.1304	374.1376	4.47	Aromatic	282.0879 135.0810
**14**	9-HPODE octadecenedioic acid	C_18_H_32_O_4_	9.80	311.223	312.2305	0.80	Fatty acid	269.2072 257.2058
**15**	9-hydroxy-10,12-octadecadienoic acid	C_18_H_32_O_3_	10.50	295.2283	296.2351	1.62	Fatty acid	269.2077
**16**	13-HPODE octadecenedioic acid	C_18_H_32_O_4_	10.56	311.2235	312.2308	3.49	Fatty acid	257.2058
**17**	(9Z,12R)-12-hydroxyoctadec-9-enoic acid	C_18_H_34_O_3_	11.52	311.2235	312.2308	5.76	Fatty acid	257.2057
**18**	Ricinoleic acid	C_18_H_34_O_3_	11.88	297.2437	298.2513	0.44	Fatty acid	253.5113
**19**	5-(octadecyloxy) isophthalic acid	C_26_H_41_O_5_	11.57	433.2866	434.2960	−21.04	Phenolic acid	
**20**	Ricinoleic acid isomer	C_18_H_34_O_3_	12.54	297.2427	298.2513	5.04	Fatty acid	283.0608 311.0561
**21**	Lesquerolic acid	C_20_H_38_O_3_	13.22	325.2745	326.2748	8.33	Fatty acid	299.2586 227.2255
**22**	Pinolenic acid	C_18_H_30_O_2_	14.23	277.2184	278.2257	4.04	Fatty acid	283.2152
**23**	9-octadecenoic acid (Z)-, 2-hydroxyethyl ester	C_20_H_38_O_3_	14.31	325.2767	326.2748	5.74	Fatty acid	307.2263 299.2586 293.2044
**24**	Roccellaric acid	C_19_H_34_O_4_	14.62	325.2396	326.2464	2.70	Fatty acid	281.2495
**25**	Alpha-boswellic acid	C_30_H_48_O_3_	14.85	455.3540	456.3613	1.37	Fatty acid	227.2255
**26**	Eicosapentaenoic acid	C_20_H_30_O_2_	15.02	301.2184	302.2257	1.79	Fatty acid	251.2660
**27**	Pristimerin	C_30_H_40_O_4_	15.02	463.2860	464.2933	1.44	Terpene	449.2673 433.2385
**28**	Dodecanoic acid,2-ethylhexanoic acid,propane-1,2,3-triol	C_23_H_48_O_7_	15.58	435.3275	436.3327	−11.95	Fatty acid	255.2610
**29**	Palmitic acid	C_16_H_32_O_2_	16.13	255.2338	256.2411	3.29	Fatty acid	112.9879
**30**	FAHFA 18:1/2:0 hydroxy fatty acid ester: icos-10-enedioic acid	C_20_H_36_O_4_	16.28	339.2553	340.2625	3.48	Fatty acid	281.2811
**31**	22-oxodocosanoic acid	C_22_H_41_O_3_	16.39	353.3116	354.3061	15.40	Fatty acid	339.2952
**31′**	PI 34:21-phosphatidyl-1D-myo-inositol	C_43_H_79_O_13_P	16.39	833.5264	834.5337	9.47	Fatty acid	717.4680 533.4085 403.2977 323.2977
**32**	Isopropyl linoleate	C_21_H_39_O_2_	16.45	323.2969	324.2956	4.12	Fatty acid	275.0326
**33**	PI(16:0/13-HODE) hydroxyoctadecadienoic acid	C_43_H_79_O_14_P	17.02	849.5204	850.5276	8.12	Fatty acid	353.3115
**34**	Muricatenol	C_37_H_68_O_6_	17.43	607.4909	607.4943	−5.49	Fatty acid	599.4280 574.4127 409.3437
**35**	28-O-acetylbetulin-3-yl-β-D-glucopyranoside	C_38_H_62_O_8_	17.52	645.4363	646.4372	−1.33	Fatty acid	607.4901 573.4062
**36**	3β-O-acetyl-28-O-lup-20(29)-ene	C_38_H_62_O_8_	17.74	645.4363	645.4367	−1.31	Sterol	409.3437
**37**	1-stearyl-2-cholesterylcarbonoyl-3-trityl glycerol	C_40_H_59_O_3_	18.56	586.4309	587.4391	−14.02	Fatty acid	281.2111
**38**	Aipolic acid	C_32_H_52_O_5_	18.51	515.3647	515.3589	11.09	Terpene	361.3160
**39**	Daucosterol	C_35_H_60_O_6_	19.12	575.4261	576.4317	−9.66	Sterol	477.3778

**Table 2:** Total phenols, flavonoids, antioxidant activity and inhibition COX-2 assays of DOe and MGEOe.

Spectrophotometric Quantification of Compounds of Interest		
Total phenols (mg GAE/g extracts)	DOe	69.24 ± 5.78
	MGEOe	65.45 ± 0.06
Flavonoids (mg QE/g extracts)	DOe	43.31 ± 4.13
	MGEOe	31.90 ± 0.05
Antioxidant and Inhibition COX-2 Assays		
DPPH (EC_50_ in µg extracts/mL)	DOe	70.11 ± 0.07
	MGEOe	62.07 ± 1.74
FRAP (mg TE/g of extracts)	DOe	4.95 ± 0.08
	MGEOe	3.25 ± 0.06
TEAC (mg TE/g of extracts)	DOe	0.67 ± 0.05
	MGEOe	1.24 ± 0.05
Percentage ILP (at 250 µg extract/mL)	DOe	83.81 ± 3.59
	MGEOe	90.46 ± 0.03
Percentage inhibition COX-2 (at 72 µg/mL)	DOe	86.97± 0.05

**Table 3:** Levels of MDA (fmol/µg of protein).

	Cerebral Cortex	Hippocampus	Hypothalamus
SUC	40.45 ± 13.82	34.67 ± 18.11	60.24 ± 7.83
HDOeS	44.39 ± 14.95	32.26 ± 7.27	46.64 ± 12.56
DOeS	46.21 ± 17.85	46.97 ± 10.65	56.20 ± 9.13

Results are presented as mean ± SD from two independent assays (*n* = 7 animals per group). The comparison between HDOeS, DOeS and SUC was performed by one-way ANOVA (F_Cerebralcortex_(2,18) = 0.248; *p* = 0.7829; F_Hippocampus_(2,18) = 2643; *p* = 0.0985 and F_Hypothalamus_(2,18) = 3.388; *p* = 0.0564).

## Data Availability

Data are contained within the article.

## Supplementary Materials

The following supporting information can be downloaded at: https://www.mdpi.com/article/10.3390/antiox13121494/s1, Gómez et al.: Table S1. BW gain (g) and average volume intake (mL/g rat); Table S2. AUC values from GTT; Table S3. Levels of BG (mg/dL), TC (g/L) and TG (g/L); Table S4. Levels of MDA (fmol/µg of protein); Table S5. OFT; Table S6. EPM, Table S7. NOL and Figure S1. MSn spectra of compounds detected by HR-MS in DOe and MGEOe (extracted using Bruker data analisis 4.0). Quinic acid (**3**); Rutin (**7**); Isovitexin (**8**); 8-hydroxy-5-methoxyflavanone (**11**); Diffractaic acid (**13**); Roccellaric acid (**24**) and Pristimerin (**27**).
