# Supplementary material for: Oxalis erythrorhiza Gillies ex Hooker et Arnott (Oxalidaceae): Chemical Analysis, Biological In Vitro and In Vivo Properties and Behavioral Effects
Source: Antioxidants (Basel). 2024 Dec 7;13(12):1494. doi: 10.3390/antiox13121494 (PMC11673872; doi:10.3390/antiox13121494)
Supplement: Supplementary file 1 [file antioxidants-13-01494-s001.zip › antioxidants-3300767-supplementary.pdf]

Table S1. BW gain (g) and average volume intake (mL/g rat).

	BW gain	Average volume intake
**CON**	328.29 ± 22.81	273.61 ± 12.82
**SUC**	334.14 ± 12.72	756.94 ± 29.07*
**HDOeA**	338.50 ± 24.24	326.49 ± 19.67
**DOeA**	367.43 ± 36.47	241.64 ± 12.03*

Results are expressed as mean ± SD from 2 independent assays (n = 7 animals/group). The groups CON and SUC were compared by t-test (BW gain: p=0.5639 and average volume intake: p<0.0001). The groups HDOeA, DOeA and CON were compared by one-way ANOVA($F_{BWgain}(2,18)=3.551$; p=0.0501 and $F_{Beverage}(2,18)=55.405$; p<0.0001) followed by Newman-Keuls' post hoc test.*p < 0.05, * refers to CON.

Table S2. AUC values from GTT

	AUC
**CON**	20406.43 ± 1425.97
**SUC**	28352.14 ± 4542.69*
**HDOeA**	24589.00 ± 4542.69
**DOeA**	22609.29 ± 2929.97

Results are expressed as mean ± SD from 2 independent assays (n = 7 animals/group). The groups CON and SUC were compared by t-test (p<0.0001). The comparisons between HDOeA, DOeA and CON were performed by one-way ANOVA ($F_{AUC}(2,18)=2.941$; p=0.0785) ollowed by Newman-Keuls' post hoc test. * p< 0.05, * refers to CON.

Table S3. Levels of BG (mg/dL), TC (g/L) and TG (g/L).

	BG	TC	TG
**CON**	99.57 ± 2.82	0.788 ± 0.069	1.469 ± 0.125
**SUC**	116.86 ± 4.02*	1.08 ± 0.09*	1.730 ± 0.142*
**HDOeA**	104.29 ± 5.28	0.802 ± 0.077	1.692 ± 0.200
**DOeA**	103.14 ± 8.17	0.885 ± 0.089	1.411 ± 0.181

Results are expressed as mean ± SD from 2 independent assays (n = 7 animals/group). The groups CON and SUC were compared by t-test (BG: p<0.0001; TC: p<0.0001 and TG: p=0.0033). The differences between HDOeA, DOeA and CON were determined by one-way ANOVA ($F_{BG}(2,18)=1.237$; p=0.3138; $F_{TC}(2,18)=3.105$; p=0.0694 and $F_{TG}(2.18)=0.976$; p=0.3957) followed by Newman-Keuls' post hoc test. * p< 0.05, * refers to CON.

Table S4. Levels of MDA (fmol/μg of protein)

	Cerebral cortex	Hippocampus	Hypothalamus
**CON**	37.31 ± 11.33	52.77 ± 18.66	39.43 ± 6.77
**SUC**	40.45 ± 13.82	34.67 ± 18.11	60.24 ± 7.83*
**HDOeA**	30.87 ± 8.55	49.59 ± 11.16	44.69 ± 10.20
**DOeA**	32.70 ± 7.59	53.58 ± 17.49	51.92 ± 10.74

Results are expressed as mean ± SD from 2 independent assays (n = 7 animals/group). The groups CON and SUC were compared by t-test (Cerebral cortex: p=0.6499; Hippocampus: p=0.0903 and Hypothalamus: p=0.0002).The comparison between HDOeA, DOeA and CON was performed by one-way ANOVA ($F_{Cerebralcortex}$(2,18)=0.891; p=0.4275; $F_{Hippocampus}$(2,18)=0.120; p=0.8877 and $F_{Hypothalamus}$(2,18)= 3.115; p=0.0689) followed by Newman-Keuls' post hoc test. * p< 0.05, * refers to CON.

Table S5. OFT

		CON	SUC	HDOeA	DOeA
**Total distance (m)**		15.90 ± 5.38	16.30 ± 5.38	15.10 ± 5.55	18.80 ± 1.87
**Lines**		144.90 ± 45.69	136.10 ± 41.81	145.00 ± 39.08	159.70 ± 20.29
**Zone 1**	Entries	6.70 ± 3.13	3.10 ± 1.45*	6.50 ± 3.66	4.40 ± 2.01
	Time (s)	10.80 ± 7.29	4.97 ± 2.89*	12.66 ± 7.22	7.32 ± 5.92
	Distance (m)	0.59 ± 0.32	0.35± 0.38	0.541 ± 0.39	0.77 ± 0.72
**Zone 2**	Entries	21.00 ± 6.27	15.60 ± 7.83	21.70 ± 6.98	16.10 ± 5.80
	Time (s)	63.80 ± 23.72	29.65 ± 12.67*	55.78 ± 21.11	68.62 ± 13.94
	Distance (m)	4.50 ± 1.58	3.00 ± 1.89	3.80 ± 1.55	3.90 ± 1.20
**Zone 3**	Entries	15.20 ± 5.96	12.60 ± 6.80	15.70 ± 4.60	12.50 ± 3.92
	Time (s)	225.19 ± 29.06	265.29 ± 12.60*	231.66 ± 23.66	224.05 ± 18.44
	Distance (m)	11.00 ± 4.60	13.10 ± 3.90	10.50 ± 4.12	14.20 ± 1.14
**Grooming**	Events	5.90 ± 3.48	3.60 ± 3.66	4.88 ± 4.26	3.34 ± 1.33
	Time (s)	12.28 ± 6.38	16.10 ± 11.32	14.44 ± 9.18	10.10 ± 8.14
**Rearing**	Events	31.23 ± 9.69	23.60 ± 7.59	22.74 ± 11.08	31.98 ± 11.35
	Time (s)	68.30 ± 21.04	56.70 ± 27.50	14.44 ± 9.18	10.10 ± 8.14

Results are expressed as mean ± SD from 2 independent assays (n = 10 animals/group). The groups CON and SUC were compared by t-test (Total distance: p=0.8698; Lines: p=0.6585; Entries zone 1: p=0.0040; Time zone 1: p=0.0302; Distance zone 1: p=0.1605; Entries zone 2: p=0.1060; Time zone 2: p=0.0008; Distance zone 2: p=0.0698; Entries zone 3: p=0.3753; Time zone 3: p=0.0008 and Distance zone 3: p=0.2850; Grooming events: p=0.1668; Grooming time: p=0.3642; Rearing events: p=0.0656 and Rearing time: p=0.3033). The comparison between HDOeA, DOeA and CON was performed by one-way ANOVA ($F_{TOTALDISTANCE}$(2,27)=1.797; p=0.1850; F_{LINES}(2,27)=0.540; p=0.5887; $F_{ENTRIESZONE1}$(2,27)=1.789; p=0.1864; $F_{TIMEZONE1}$(2,27)=1.571; p=0.2262; $F_{DISTANCEZONE1}$(2,27)=0.578; p=0.5676; $F_{ENTRIESZONE2}$(2,27)=2.296;

p=0.1200; $F_{TIMEZONE2}(2,27)=1.049$; p=0.3640; $F_{DISTANCEZONE2}(2,27)=0.679$; p=0.5156; $F_{ENTRIESZONE3}(2,27)=1.234$; p=0.3069; $F_{TIMEZONE3}(2,27)=0.290$; p=0.7508 and $F_{DISTANCEZONE3}(2,27)=3.073$; p=0.0628; $F_{Groomingevents}(2,24)=1.498$; p=0.2438; $F_{Groomingtime}(2,24)=0.644$; p=0.5338) and $F_{Rearingevents}(2,27)=2.288$; p=0.1208 and $F_{Rearingtime}(2,27)=3.137$; p=0.0596) followed by Newman-Keuls' post hoc test.* p< 0.05, * refers to CON. It is important to note that only 8 and 9 animals from the groups HDOeA and DOeA, respectively, performed the grooming activity.

Table S6. EPM

		**CON**	**SUC**	**HDOeA**	**DOeA**
**Closed arms**	Entries	14.76 ± 4.86	15.70 ± 5.43	15.83 ± 7.34	13.69 ± 7.81
	Time (s)	166.51 ± 46.77	161.50± 31.90	137.46 ± 51.81	189.74 ± 49.21
	Distance (m)	5.56 ± 1.89	6.19 ± 1.87	4.64 ± 1.57	6.19 ± 2.16
**Open arms**	Entries	8.22 ± 5.49	7.85 ± 4.00	5.61 ± 2.06	7.34 ± 5.55
	Time (s)	60.16 ± 41.38	60.70 ± 22.34	72.24 ± 35.61	50.13 ± 37.62
	Distance (m)	1.69 ± 1.25	1.73 ± 0.82	2.04 ± 1.27	1.28 ± 1.06
**Center**	Entries	21.88 ± 9.68	22.14 ± 8.13	19.25 ± 8.42	19.90 ± 6.42
	Time (s)	73.30 ± 29.69	77.94 ± 19.34	90.29 ± 37.33	60.19 ± 12.54
	Distance (m)	0.93 ± 0.27	1.31 ± 0.62	0.94 ± 0.28	0.97 ± 0.40
**Grooming**	Events	2.78 ± 2.05	2.00 ± 0.82	3.40 ± 2.30	5.13 ± 2.60
	Time (s)	12.33 ± 10.82	13.04 ± 10.62	17.20 ± 10.50	14.50 ± 5.81
**Rearing**	Events	12.20 ± 5.63	14.70 ± 4.60	9.70 ± 4.90	13.40 ± 3.69
	Time (s)	34.10 ± 12.85	30.82 ± 11.44	23.30 ± 10.79	24.10 ± 9.36
**Head dips events**		9.70 ± 4.76	12.10 ± 4.80	9.60 ± 2.55	10.30 ± 5.83

Results are expressed as mean ± SD from 2 independent assays (n = 10 animals/group). The groups CON and SUC were compared by t-test (Closed arms entries: p=0.6885; time: p=0.7828 and distance: p=0.4630; Open arms entries: p=0.8647; time: p=0.9714 and distance: p=0.8934; and Center entries: p=0.9484; time: p=0.6839 and distance: p=0.0912; Grooming events: p=0.3620; Grooming time: p=0.9039; Rearing events: p=0.2912; Rearing time: p=0.5516 and Head dips events (p=0.2762). It is important to note that only 9 and 7 animals from the groups CON and SUC, respectively, performed the grooming activity. The comparison between HDOeA, DOeA and CON was performed by one-way ANOVA in Closed ($F_{Entries}(2,27)=0.249$; p=0.7813; $F_{Time}(2,27)=2.822$; p=0.0771 and $F_{Distance}(2,27)=1.702$; p=0.2013) and Open arms ($F_{Entries}(2,27)=0.811$; p=0.4551; $F_{Time}(2,27)=0.837$; p=0.4439 and $F_{Distance}(2,27)=1.011$; p=0.3773), Center ($F_{Entries}(2,27)=0.274$; p=0.7626; $F_{Time}(2,27)=2.810$; p=0.0779 and $F_{Distance}(2,27)=0.048$; p=0.9530), Grooming ($F_{Events}(2,19)=2.265$; p=0.1311 and $F_{Time}(2,19)=0.454$; p=0.6418), Rearing ($F_{Events}(2,27)=1.542$; p=0.2323 and $F_{Time}(2,27)=2.943$; p=0.0698) and Head dips events ($F_{Events}(2,27)=0.068$; p=0.9344) followed by Newman-Keuls' post hoc test. It is important to note that only 5 and 8 animals from the groups HDOeA and DOeA, respectively, performed the grooming activity.

Table S7. NOL

	ER	LINE CROSSING		TIME IN T1	
		**T1**	**T2**	**OBJECT N**	**OBJECT F**
**CON**	0.652 ± 0.079	151.56 ± 44.16	151.56 ± 44.16	29.34 ± 12.28	27.61 ± 9.13
**SUC**	0.421 ± 0.078*	122.00 ± 28.90	122.00 ± 28.90	28.30 ± 10.15	28.02 ± 7.97
**HDOeA**	0.569 ± 0.079	106.7 ± 42.22	106.7 ± 42.22	34.17 ± 12.02	26.72 ± 5.83

Results are expressed as mean ± SD from 2 independent assays (n = 7 animals/group). The groups CON and SUC were compared by t-test (lines in T1: p=0.1642 and T2: p=0.0607; time in T1 for object N: p=0.8658 and object F: 0.9304; and ER: p=0.0001). The comparison between HDOeA and CON were performed by t-test (lines in T1: p=0.0759 and T2: p=0.0514; time in T1 for object 1: p=0.4712 and object 2: 0.8311; and ER: p=0.0717). * $p < 0.05$, * refers to CON. It is important to note that the DOeS group was excluded because only three animals explored both objects on the trials for the time required for the analysis.

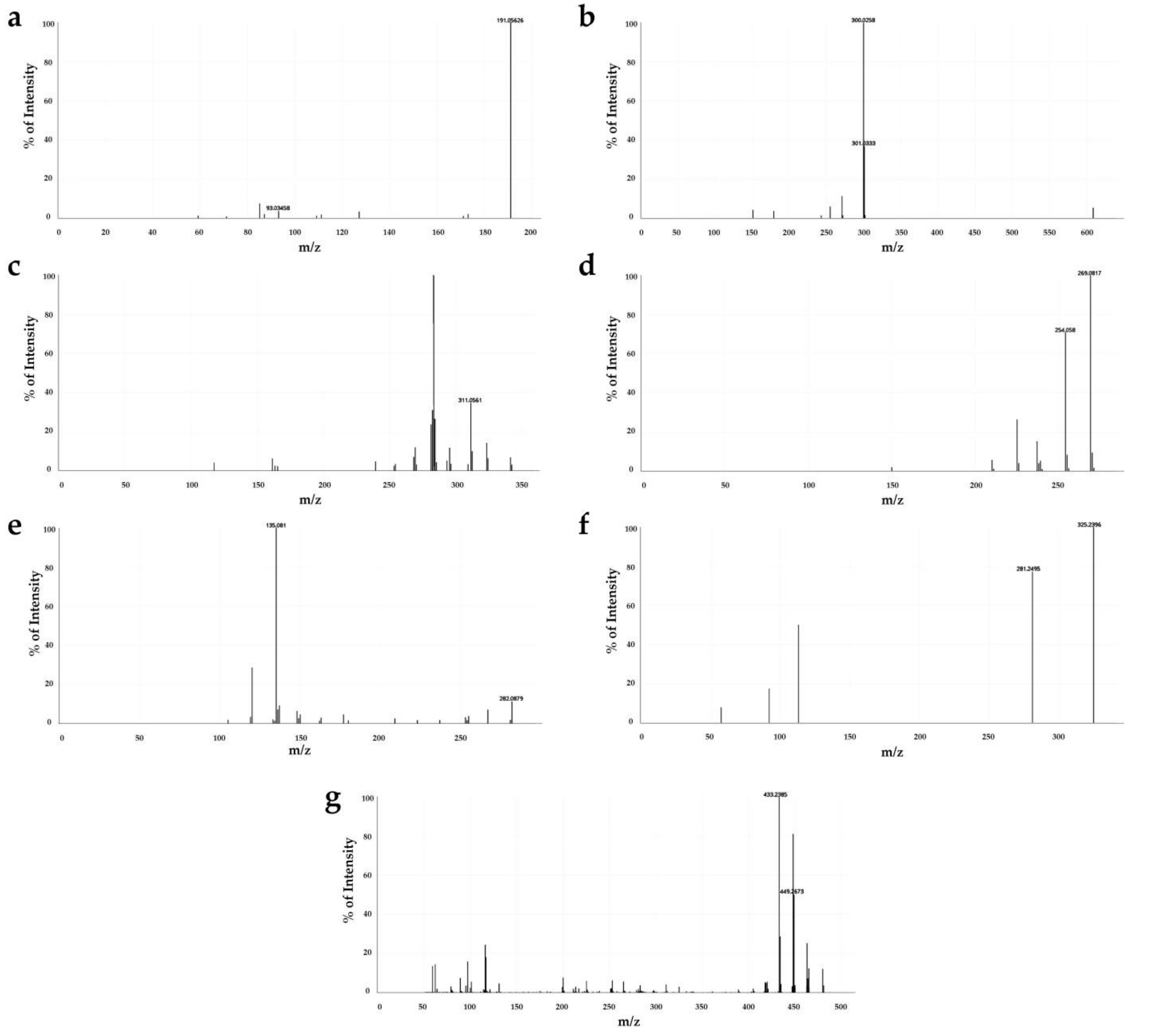

Figure S1: MSn spectra of compounds detected by HR-MS in DOe and MGEOe (extracted using Bruker data analisis 4.0). **a**) Quinic acid (**3**); **b**) Rutin (**7**); **c**) Isovitexin (**8**); **d**) 8-hydroxy-5-methoxyflavanone (**11**); **e**) Diffractaic acid (**13**); **f**) Roccellaric acid (**24**) and **g**) Pristimerin (**27**).
